# Radiomics biopsy signature for predicting survival in patients with spinal bone metastases (SBMs)

**DOI:** 10.1016/j.ctro.2021.12.011

**Published:** 2022-01-05

**Authors:** I. Sanli, B. Osong, A. Dekker, K. TerHaag, S.M.J. van Kuijk, J. van Soest, L. Wee, P.C. Willems

**Affiliations:** a Department of Orthopedic Surgery, Maastricht University Medical Center, The Netherlands; b Department of Radiation Oncology (MAASTRO), GROW - School for Oncology and Developmental Biology, Maastricht University Medical Center, The Netherlands; c Department of Clinical Epidemiology and Medical Technology Assessment (KEMTA), Maastricht University Medical Center, Maastricht, The Netherlands

**Keywords:** Spinal bone metastases, SBM, Radiomics, Predictive model

## Abstract

**Study design:**

Retrospective analysis of a registered cohort of patients treated and irradiated for metastases in the spinal column in a single institute.

**Objective:**

This is the first study to develop and internally validate radiomics features for predicting six-month survival probability for patients with spinal bone metastases (SBM).

**Background data:**

Extracted radiomics features from routine clinical CT images can be used to identify textural and intensity-based features unperceivable to human observers and associate them with a patient survival probability or disease progression.

**Methods:**

A study was conducted on 250 patients treated for metastases in the spinal column irradiated for the first time between 2014 and 2016, at the MAASTRO clinic in Maastricht, the Netherlands. The first 150 available patients were used to develop the model and the subsequent 100 patient were considered as a test set for the model. A bootstrap (B = 400) stepwise model selection, which combines both the forward and backward variable elimination procedure, was used to select the most useful predictive features from the training data based on the Akaike information criterion (AIC). The stepwise selection procedure was applied to the 400 bootstrap samples, and the results were plotted as a histogram to visualize how often each variable was selected. Only variables selected more than 90 % of the time over the bootstrap runs were used to build the final model.

A prognostic index (PI) called radiomics score (radscore) and clinical score (clinscore) was calculated for each patient. The prognostic index was not scaled, the original values were used which can be extracted from the model directly or calculated as a linear combination of the variables in the model multiplied by the respective beta value for each patient.

**Results:**

The clinical model had a good discrimination power. The radiomics model, on the other hand, had an inferior performance with no added predictive power to the clinical model. The internal imaging characteristics do not seem to have a value in the prediction of survival. However, the Shape features were excluded from further analyses in our study since all biopsies had a standard shape hence no variability.

## Highlights

• Prediction of survival is crucial for guiding patient-tailored treatment.
• Radiomics can be described as the next era of possibilities in precision medicine.
• Radiomics model had an inferior performance with no added predictive power to the clinical predictive model.

## Introduction

Spinal bone metastases (SBMs) are often accompanied by a significant burden of morbidity, causing cancer-induced bone pain, pathologic fractures, or neurological complications as a consequence of nerve root and spinal cord compression, leading to a reduced quality of life and impaired survival [1]. An accurate estimation of survival is required to prevent invasive surgery in patients with only a short-term survival expectancy and to prevent the omission of treatment in patients with a more prolonged survival. Two systematic reviews showed that physicians’ assessment of life expectancy based solely on their clinical experience is inaccurate [2], [3], [4]. Controversies often exist between the best clinical practices determined by scientific evidence and the actual care provided to patients; about 30–40 % of patients do not receive care based on the current scientific evidence, and about 20–25 % of the care provided is unnecessary or even potentially harmful to patients [5]. Hence, prediction of prognosis is crucial for counselling patients and for selecting the most adequate treatment for a patient, thus ensuring appropriate allocation of health care resources. Several studies have been published to assess the prognostic value of single variables, and multiple variables combined into predictive models. However, existing predictive models lack discriminative ability, particularly predicting which patients will survive for more than 3 to 6 months and become potential candidates for surgical treatment [5], [6], [7], [8], [9], [10], [11], [12], [13], [14], [15]. Therefore, there’s a significant need for new prognostic biomarkers. Tissue markers derived from tumor biopsies usually represent only a small tumor subregion at a single time point. Therefore, they are often not representative of the tumors’ biology or the biological alterations during and after treatment. Radiomics has the potential to give complete three-dimensional tumor information. Radiomics, which extracts and analyses vast amounts of advanced quantitative imaging features with high throughput from medical images like Computed Tomography (CT), is gaining interest in health care and becoming increasingly important [16].

The analyses of Big Data (Omics) allows us to define biomarker signatures, which may significantly improve the prediction of outcomes [17]. Extracted radiomics features from routine clinical CT images can be used to train a machine-learning prediction model to identify textural and intensity-based features unperceivable to human observers and associate them with a patient survival probability or disease progression. Furthermore, these predicted probabilities can be used to classify patients into risk categories for more precise and timely therapeutic interventions. These non-invasive techniques for guiding treatment decisions could complement the present conventional methods. And with our increasing knowledge of cancer biology, these techniques could play an essential role in the future of cancer treatment.

The aim of this study was to develop and internally validate radiomics features in a predictive model. Can the use of (current) radiomics help improve the prediction of survival as based on clinical features in SBM patients?

## Materials & methods

### Patients

A retrospective study was conducted on 250 patients treated for metastases in the spinal column irradiated for the first time between January 1, 2014, and December 31, 2016, at the MAASTRO clinic in Maastricht, the Netherlands. The first 150 available patients were used to develop the model and the subsequent 100 patient were considered as a test set for the model. Of the 100 patients included in the test data, 13 (13 %) had no images reducing the test data set to 87 patients. The following patient characteristics were considered for their prognostic value for predicting survival: age, gender, primary tumor type metastasis, location treated spinal metastases causing symptoms, radiation field, radiotherapy fractionation schedule, pathological fracture, spinal compression, lymphatic metastases, pain score, visceral metastases, brain metastases, World Health Organization (WHO) performance score. The primary tumors were categorized based on the classification used by Bollen et al. [11]. In the original Tomita classification, growth speed alone was used to assign a primary tumor into 1 of 3 groups [6]. Bollen renamed the classification “clinical profile” to encompass other contributing factors such as the availability of effective systemic treatment options for the primary tumor. The clinical profile of a primary tumor was considered to be favorable, moderate, or unfavourable [11]. These variables were complemented with SBM tumor characteristics by the use of Radiomics analysis.

### Feature extraction and processing

One physician (IS) and a physician assistant (KtH) independently segmented the regions of interest by taking multiple (5 to 10) “virtual” biopsies (A small portion of the ROI that is large enough to capture the heterogeneity of the tumor) of 1 cm in diameter from the obtained CT scans. Seven feature classes were extracted using the Ontology-guided Radiomics Analysis Workflow (O-RAW) version 2.0 software (https://gitlab.com/UM-CDS/o-raw)

• **Shape**
• **First-order**
• **Texture:**
  o Gray Level Dependence Matrix (**GLDM**)
  o Gray Level Size Zone Matrix (**GLSZM**)
  o Gray Level Co-occurrence Matrix (**GLCM**)
  o Gray Level Run Length Matrix (**GLRLM**)
  o Neighboring Gray Tone Difference Matrix (**NGTDM**)

The Shape features were excluded from further analyses since all biopsies had a standard shape hence no variability. To ensure reproducibility, the intra-class correlation coefficient (ICC), which evaluates the degree of agreement and correlation between measurements, was used to assess the stability and robustness of the extracted radiomics feature values between the two physicians (ICC < 0.50, low agreement; 0.50 ≤ ICC < 0.80, median agreement; ICC ≥ 0.80, high agreement). The maximum value of ICC is 1, which indicates perfect agreement. The lower the ICC, the lower the similarity among the features extracted values between the two physicians. Only features with an ICC > 0.8 were considered for subsequent analyses.

### Feature selection and signature building

A bootstrap (B = 400) stepwise model selection, which combines both the forward and backward variable elimination procedure, was used to select the most useful predictive features from the training data based on the Akaike information criterion (AIC). Only variables selected more than 90 % of the time over the bootstrap runs were used to build the final model. A prognostic index (PI) called radiomics score (radscore) and clinical score (clinscore) was calculated for each patient via a linear combination of the selected features and weighted by their respective regression coefficients for a practical application. Higher values for these scores indicate a poorer prognosis for the patients’ survival outcomes.

### Statistical analysis

**Fig. 1:**
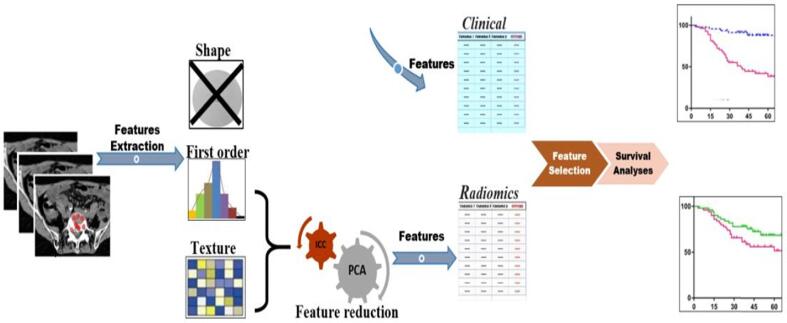
Analyses scheme for building the spinal metastases models to predict six months’ survival using radiomics biopsy and clinical information.

Exploratory data analysis (EDA) and principal component analysis (PCA) were performed to detect abnormal patterns and possible outliers within the data. Survival time was defined as the difference between the start of treatment for the spinal metastasis and the date of death or last follow-up record. Those patients alive at the end of their follow-up were censored. Cox proportional hazard regression models were fitted to evaluate the performance of the selected clinical and radiomic predictors. Harrell’s C statistic, which estimates the probability of concordance between predicted and observed responses, was used to validate the models’ predictive value. Survival curves were estimated using the Kaplan-Meier method, and log-rank tests were used to compare the differences in survival curves. A p-value <0.05 was considered statistically significant. The Z-score transformation was applied to have the radiomics features on the same scale. Fig. 1 shows the analysis schema for this study.

### Software packages

Statistical analysis, model training, validation, and visualization were performed in R version 3.6.1.

## Results

The majority of the patients in the study were males 135 (57 %), and the median age (range) of all patients was 68 years (24–92 years) (Table 1). There was no statistically significant difference between patients who were alive and those who died for almost all the variables for both the train and test data, except for the variables clinical profile and visceral metastases. The pain score variable was excluded from the analyses because of the high percentage of missing values.

**Table 1:** Detailed characteristic of the studied cohorts.

Characteristic	Train on 150			Validate on 87		
	Dead	Alive	p-value	Dead	Alive	p-value
Age at RT in years [mean (Min-Max)]	67 (24–92)	68 (46–88)	0.524	72 (50–88)	67 (39–86)	0.041
Sex						
Male	39 (48.8 %)	41 (51.2 %)	0.844	31 (56.4 %)	24 (43.6 %)	0.392
Female	33 (47.1 %)	37 (52.9 %)		15 (46.9 %)	17 (53.1 %)	

WHO performance score						
Restricted	28 (40.0 %)	42 (60.0 %)	0.174	13 (46.4 %)	15 (53.6 %)	0.215
Self-care	29 (50.0 %)	29 (50.0 %)		17 (47.2 %)	19 (52.8 %)	
Limited Self-care	14 (66.7 %)	7 (33.3 %)		16 (72.7 %)	6 (27.3 %)	
*Missing*	1 (100 %)	0(0.0 %)		0 (0.0 %)	1 (100 %)	

Clinical profile						
Favorable	3 (8.8 %)	31 (91.2 %)	<0.005	4 (28.6 %)	10 (71.4 %)	0.021
Moderate	15 (37.5 %)	25 (62.5 %)		11 (42.3 %)	15 (57.7 %)	
Unfavorable	54 (71.1 %)	22 (28.9 %)		31 (66.0 %)	16(34.0 %)	

Location treated spinal metastases						
Diffuse	22 (66.7 %)	11 (33.3 %)	0.212	6 (46.2 %)	7 (53.8 %)	0.692
Cervical	4 (40.0 %)	6 (60.0 %)		2 (33.3 %)	4 (66.7 %)	
Lumbar	23 (40.4 %)	34 (59.6 %)		15 (57.7 %)	11 (42.3 %)	
Thoracic	23 (46.0 %)	27 (54.0 %)		23 (54.8 %)	19 (45.2 %)	

Number of spinal metastases						
1	15 (45.5 %)	18 (54.5 %)	0.486	10 (66.7 %)	5 (33.3 %)	0.308
2	17 (41.5 %)	24 (58.5 %)		07 (63.6 %)	04 (36.4 %)	
3 or more	40 (52.6 %)	36 (47.4 %)		29 (47.5 %)	32 (52.5 %)	

# of extra spinal bone metastases						
None	23 (46.9 %)	26 (53.1 %)	0.376	6 (60.0 %)	4 (40.0 %)	0.883
1 or 2	13 (61.9 %)	8 (38.1 %)		5 (50.0 %)	5 (50.0 %)	
3 or more	36 (45.0 %)	44 (55.0 %)		35 (52.2 %)	32 (47.8 %)	

Visceral metastases						
Present	32 (59.3 %)	22 (40.7 %)	0.038	26 (66.7 %)	13 (33.3 %)	0.020
Not present	40 (41.7 %)	56 (58.3 %)		20(41.7 %)	28 (58.3 %)	

Brain metastases						
Present	9 (100 %)	0 (0.0 %)	0.001	2 (100 %)	0 (0.0 %)	0.176
Not present	63 (44.7 %)	78 (55.3 %)		44 (51.8 %)	41 (48.2 %)	

Pain score						
No pain	1 (33.3 %)	2 (66.7 %)	0.251	1 (33.3 %)	2 (66.7 %)	0.784
Mild	2 (28.6 %)	5 (71.4 %)		2 (66.7 %)	1 (33.3 %)	
Moderate	16 (64.0 %)	9 (36.0 %)		6 (60.0 %)	4 (40.0 %)	
Severe	11 (45.8 %)	13 (54.2 %)		10(66.7 %)	5 (33.3 %)	
Very severe	19 (48.7 %)	20 (51.3 %)		10 (43.5 %)	13 (56.5 %)	
Worst possible	6 (75.0 %)	2 (25.0 %)		2 (40.0 %)	3 (60.0 %)	
*Missing*	17 (38.6 %)	27 (61.4 %)		15 (53.6 %)	13 (46.4 %)	

Pathological fracture						
Yes	15 (50.0 %)	15 (50.0 %)	0.806	14 (53.8 %)	12 (46.2 %)	0.906
No	57 (47.5 %)	63 (52.5 %)		32(52.5 %)	29 (47.5 %)	

Spinal compression						
Yes	8 (28.6 %)	20 (71.4 %)	0.022	12 (54.5 %)	10 (45.5 %)	0.856
No	64 (52.5 %)	58 (47.5 %)		34 (52.3 %)	31 (47.7 %)	

lymphatic metastases						
Present	32 (53.3 %)	28 (46.7 %)	0.286	24 (53.3 %)	21 (46.7 %)	0.929
Not present	40 (44.4 %)	50 (55.6 %)		22 (52.4 %)	20 (47.6 %)	

RT: Radiotherapy, #: Number.

The interobserver agreement of the extracted features was good (Table 2). Hence, the median biopsy radiomics value for each patient was considered in this study. The first radiomic feature reduction process, which considered only features with an ICC value above 0.8 and the exclusion of shape features, reduced the radiomics feature from 105 to 19. Two patients, one with a missing WHO performance score (Table 1) and another with extreme outlying value (Fig. 1, supplementary material) due to artifacts on the image, were excluded reducing the total training sample size to 148. The stepwise selection procedure selected three radiomics features (glszm Small Area Emphasis, gldm Small Dependence Emphasis, gldm Dependence Non-Uniformity Normalized) and two clinical features (Clinical profile and WHO performance score) as shown in Fig. 1, supplementary material. The median follow-up time was 22.37 (95 % CI: 10.22–36.14) and 15.21 (95 % CI: 9.79–20.60) months for the training and testing data, respectively.

**Table 2:** Inter-observer analysis, showing the ICC values and the number of stable features per feature group, defined as high (ICC ≥ 0.8), median (0.8 > ICC ≤ 0.5), and low (ICC < 0.5) stability.

*N-^0^*	Stability class	N	ICC	ICC (95 % CI)
1	First order statistics			
	High stability	8	0.810	0.795–0.823
	Medium stability	8	0.510	0.478–0.540
	Low stability	1	0.330	0.292–0.366

2	Gray Level Co-occurrence Matrix (GLCM)			
	High stability	3	0.820	0.805–0.833
	Medium stability	13	0.500	0.468–0.530
	Low stability	6	0.240	0.200–0.278

3	Gray Level Run Length Matrix (GLRLM)			
	High stability	1	0.810	0.795–0.823
	Medium stability	7	0.52	0.488–0.549
	Low stability	8	0.240	0.200–0.278

4	Gray Level Size Zone Matrix (GLSZM)			
	High stability	5	0.810	0.795–0.823
	Medium stability	7	0.54	0.509–0.568
	Low stability	4	0.24	0.200–0.278

5	Gray Level Dependence Matrix (GLDM)			
	High stability	2	0.820	0.805–0.833
	Medium stability	6	0.680	0.656–0.701
	Low stability	6	0.240	0.200–0.278

6	Neighbouring Gray Tone Difference Matrix (NGTDM)			
	High stability	1	0.800	0.784–0.814
	Medium stability	4	0.500	0.468–0.530
	Low stability	–	–	–

**Fig. 2:**
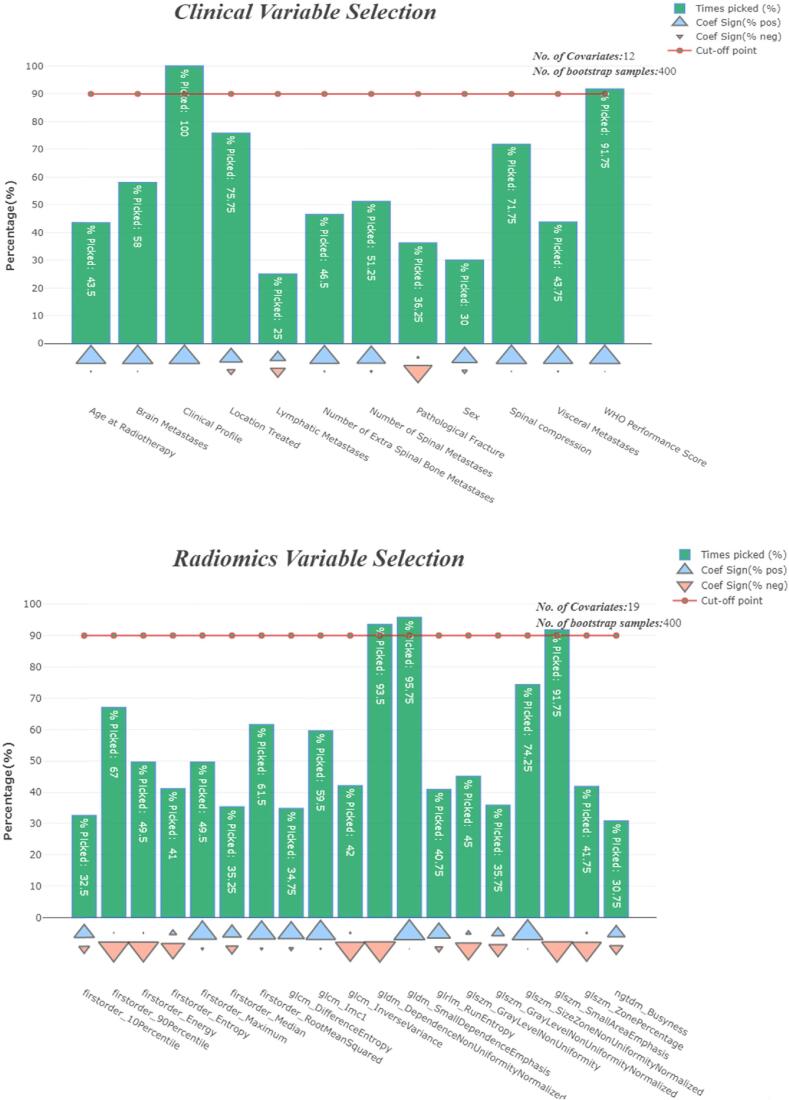
Bootstrap (B = 400) stepwise variable selection procedure for the clinical and radiomics data. The green bars show the percentage of time a variable was selected. The blue and red triangles (Coef Sign) show a represented rate of times the variable’s coefficient was positive or negative in each bootstrap run, respectively. The horizontal line shows the cut-off point for selected variables. (For interpretation of the references to colour in this figure legend, the reader is referred to the web version of this article.)

The three radiomic features and two clinical features selected by the stepwise procedure in the training dataset were used to compute the radscores- and clinscores. The proportional hazards assumption was supported since there was a non-significant relationship between scaled Schoenfeld residuals and time. The plot of the scaled Schoenfeld residuals against the transformed time also had no pattern (Fig. 2, supplementary material).

Table 3 shows the univariable and multivariable performance of the scores in the training and testing data. As observed from the table, both scores are significant independent prognostic factors for six months survival in the train data with a p-value <0.05. However, the discriminating power of the radscore model was lower than the clinscore model with a C-index of 0.623 (95 % CI: 0.553–0.693). The clinscore models, on the contrary, had a relatively better discriminating power with a C-index of 0.731 (0.682–0.801). Based on the results of multivariable analysis, both scores were still significantly associated with the outcome (p-value < 0.05), but with a C-index of 0.740 (0.686–0.794), which is an indication that the radiomics model adds little or no information to the clinical model.

**Table 3:** Univariate and multivariate predictive performance of the scores.

Variables	Training Data		Testing data
	C-index (95 % CI)	p-value	C-index (95 % CI)
*Univariate scores*			
RadScore	0.623 (0.553–0.693)	<0.05	0.570 (0.497–0.642)
ClinScore	0.731 (0.682–0.801)	<0.05	0.686 (0.602–0.770)

*Multivariate scores*			
RadScore	0.740 (0.686–0.794)	0.01	0.669 (0.598–0.740)
ClinScore		<0.05	

The clinscore still had a decent discriminating power in the test data, but with a slightly low C-index of 0.686 (0.602–0.770) compared to the train data. The radscore, on the other hand, had a poor performance with a C-index of 0.570 (0.497–0.642), which is only slightly better than a random guess. The multivariable model with both scores shows that the addition of the radscore negatively affected the model’s discriminating power with a reduced C-index value of 0.669 (0.598–0.740), which might indicate overfitting.

**Fig. 3:**
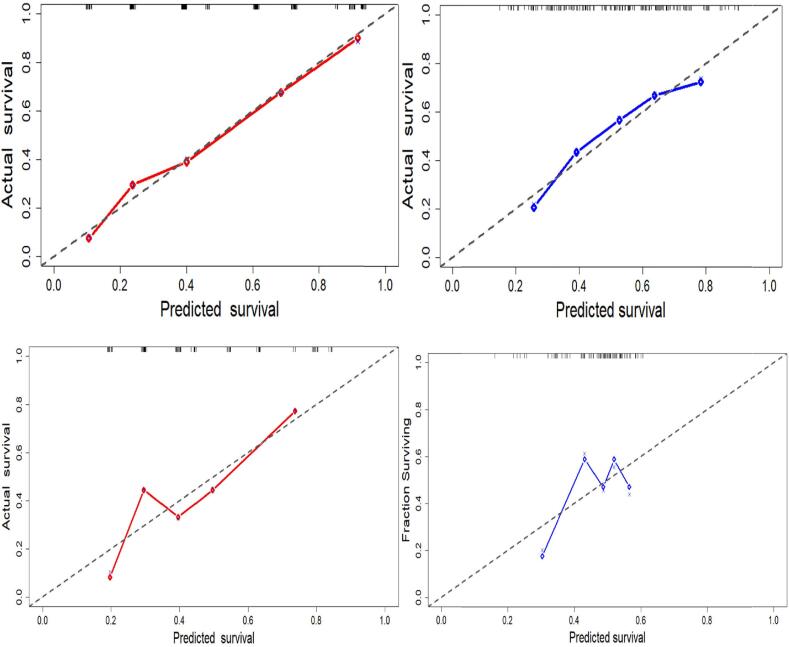
Calibration plots for clinscore and radscore, respectively, for the train(top) and test(bottom) data. The predicted survival is plotted on the x-axis, and the actual survival is plotted on the y-axis. The dotted gray line represents an ideal fit where the predicted probabilities perfectly match the observed probabilities. The diamonds show the estimated model performance, and the crosses indicate bias-corrected estimates.

The calibration plot, which measures the similarities between the observed and predicted probabilities, was used to evaluate further the performance of the score models in the training and testing data. The closer the points are to the diagonal dotted line, the more accurate the model predicts the outcome. Fig. 3 show that the model is well calibrated on the train data, especially for clinscore. However, the model looks less well-calibrated on the test data, especially the radscore with its point falling far from the diagonal line.

**Fig. 4:**
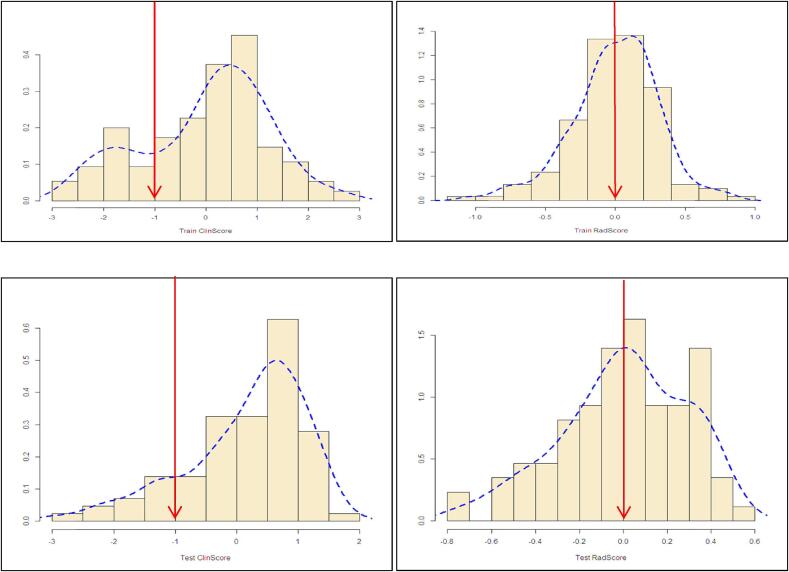
Histogram of the clinscore and radscore in the train and test datasets respectively. The red arrows indicates the optimal cut-off point used to categorize the patients into a low and high risk groups in each dataset. (For interpretation of the references to colour in this figure legend, the reader is referred to the web version of this article.)

The scores values were categorized to separate the patient into two risk groups based on some cut-off values determined from the frequency distribution of the scores as shown on the histogram plot (Fig. 4). The chosen cut-off scores used for separating the patients into high (>cut-off) and low (≤cut-off) risk groups from the train data were translated to the test data. The clinscore had a bimodal distribution; hence a cut-off value of −1, which separates the two distributions, was chosen. For the radscore, which had a normal distribution, the median value of 0.044 was chosen.

**Fig. 5:**
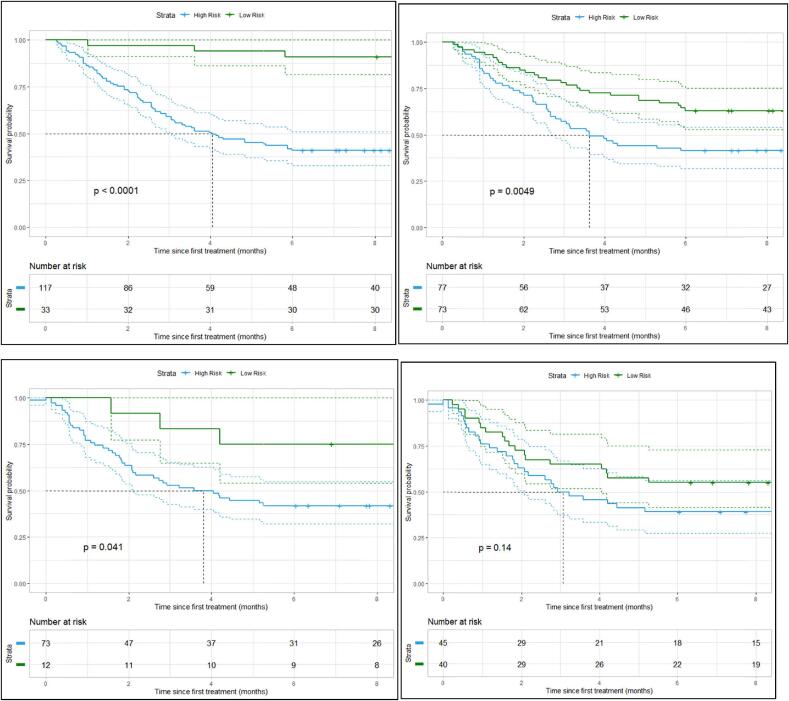
Kaplan-Meier curves for six months’ survival in the low and high-risk groups based on the cut-off points in the clinscore and radscore for the train (top) and test (below) datasets, respectively.

Furthermore, stratification analyses based on the risk groups showed that both scores were still independent predictors in discriminating the survival of SBM patients with a p-value <0.05 in the train data. In the test data, no statistical significance survival difference was observed between the two radscore groups with a p-value of 0.14, suggesting that the radscore might be slightly over-fitted to the train data. However, there was a borderline significance difference (p-value 0.04) between the two clinscore risk groups (Fig. 5).

## Discussion

The number of people in society diagnosed with cancer is increasing. Additionally, survival of patients with cancer is extended because of improved treatment options, thus allowing for the emergence of more metastases.^2^ The spinal column is a common site of metastatic disease. In autopsy studies, up to 90 % of patients with cancer, metastatic deposits are observed, of which approximately 30 % of patients will be symptomatic. Adult patients with cancer of the lung, breast, and prostate are most likely to be affected [18].

For patients with SBMs, the primary goals of treatment should be focused on quality of life. Prediction of survival is crucial for guiding the appropriate choice of treatment (patient-tailored treatment). Numerous tools have been established to predict individual patient’s survival and propose an appropriate corresponding therapeutic strategy. External validation studies, however, demonstrated confusing inconsistency between predicted and actual survival [19], [20], [21].

In the retrospective study of Bollen et al. in which 1043 patients were treated for symptomatic SBMs, only clinical profile of the primary tumour, performance status, and in the subgroup of favourable clinical profile, the presence of visceral and brain metastases was associated with survival. Van der Linden et al. showed in their prospective randomized radiotherapy trial that primary tumor, Karnofsky performance score, and absence of visceral metastases were significant predictors in the survival of patients with painful SBMs. In our study, only two prognostic factors showed significant association with survival, that is clinical profile, and the WHO performance status. The presence of visceral metastasis and clinical profile of the patient were the only predictors with a statistically significant difference between SBM survivors and no-survivors in both the training and testing data, although visceral metastasis was not selected. However, the predictive value of visceral metastasis for survival in patients with spinal metastases is controversial in current literature [22], [23]. A recent meta-analysis suggested that the occurrence of visceral metastases has a strong negative impact on survival and should be considered when choosing a precision treatment [24]. Interestingly, the presence of visceral metastases exhibited various impacts on survival in different primary tumors. However, visceral metastasis in thyroid, breast and renal cancer could not yet be confirmed as a significant prognostic factor for survival. Large prospective trials are required to define better the prognostic value of visceral metastasis in a patient with different tumors. In our study, the clinscore models showed a good discrimination power with a C-index of 0.73. There seems to be a role for specific clinical factors in survival prediction. However, the number of patients in our training and test set was low. Ideally, with higher numbers, we might have better performance with a smaller chance of overfitting.

In clinical practice, invasive biopsy and molecular assays are needed to specify tumors. However, spatial and temporal pathologic heterogeneity limits the ability of one-moment invasive biopsies to capture their biological diversity or disease evolution. Furthermore, repeated invasive tumor sampling can be troublesome, expensive, and limited by the practical number of tissue sampling that can be undertaken to monitor disease progression or treatment response. By contrast, the non-invasive imaging phenotype potentially contains a treasure of information that can inform on the expression of the genotype, the tumor microenvironment, and the susceptibility of the tumor to treatment.

Radiomics can be described as the next era of possibilities in precision medicine. An emerging research field aiming to find associations between qualitative and quantitative information extracted from clinical images and clinical data, to support evidence-based clinical decision-making. Different kinds of features can be derived from clinical images. Quantitative features are usually categorized into the following subgroups [25]. Shape features describing the shape of the traced region of interest (ROI) and its geometric properties. First-order statistics features describe the distribution of individual voxel values without concern for spatial relationships. Second-order statistics features are obtained, calculating the statistical interrelationships between neighboring voxels. They provide a measure of the spatial arrangement of the voxel intensities and hence of intra-lesion heterogeneity. Higher-order statistics features are obtained by statistical methods after applying filters or mathematical transforms to the images.

In this paper, we studied the predictive value of first-order and texture radiomics signatures. We found no added discriminative effect of the studied radiomics signatures. So the internal imaging characteristics do not seem to have a value in the prediction of survival. However, the Shape features were excluded from further analyses in our study since all biopsies had a standard shape hence no variability. Especially volume seems to predict well in many Radiomics analyses. A study by Roy et al. found that of all radiomic features tested in their study, 16 were found to be volume-dependent [26]. Their evidence indicates that tumor volume significantly impacts radiomic features in co-clinical imaging, in which they propose a volume-dependency correction scheme and identify a set of robust radiomic features for co-clinical imaging studies.

A major strength of a radiomics approach for cancer is that digital radiologic images are obtained for almost every patient with cancer, and all of these images are potential sources for radiomics databases. It is conceivable that the lack of quantitative information leads to increased follow-ups or invasive biopsies that would be deemed unnecessary given the unused information in medical images. Besides features encode morphological information beyond the limits of the human eye. When the feature extraction is performed expertly, artificial intelligence trained on handcrafted radiomics features can perform as deep learning, especially in smaller data sets.

However there are some other critical comments which can be made. Algorithms contain human bias and delineation of hand crafted radiomics features is time consuming. Besides routine clinical imaging techniques show a wide variation in acquisition parameters, such as image spatial resolution; administration of contrast agents; kVp and mAs (among others) for CT; type of sequence, echo time, repetition time, number of excitations, and many other sequence parameters for MRI. Furthermore, different vendors offer different reconstruction algorithms, and reconstruction parameters are customized at each institution, with possible variations in individual patients. All these variables affect image noise and texture, and consequently, radiomic features. Standard CT phantoms, allow the evaluation of imaging performance and the assessment of how far image quality depends on the adopted technique. Despite not being intended for this, they may provide useful information on the parameters potentially affecting image texture. Segmentation is another critical step of the radiomics process because data are extracted from the segmented volumes. This is challenging because many tumors show unclear borders, and the reproducibility of the segmentation is questionable. Hence radiomic features are susceptible to image acquisition and segmentation variability. Ideally, only features robust to these variations would be incorporated into predictive models for good generalizability or a reproducible, automated algorithm for segmentation should be used. Other factors such as the presence of artifacts due to metallic prostheses, may affect image quality and impair quantitative analysis. Furthermore, electronic density quantification expressed as Hounsfield Units may vary with the reconstruction algorithm or scanner calibration.

Radiomics is a growing field based on the analysis of hand-crafted features, which depend on an arbitrary decision to apply a statistical analysis to an image as a form of feature engineering. Deep learning can extract learned features from images which may be more helpful in determining the required outcome. Combining the learned features extracted via deep learning and the current hand-crafted radiomic features may possibly improve outcome prediction. Deep learning combined with machine learning has the potential to advance the Radiomics field, provided the raw data is available for the results to be determined robustly across all patient and tumor types [27].

## Conclusions

We have developed and validated a clinical and Radiomics model for predicting six-month survival probability for patients with SBM. The clinical model had a good discrimination power. The radiomics model, on the other hand, had an inferior performance with no added predictive power to the clinical model, which might be due to the excluded shape feature. Therefore using a more sophisticated approach like deep learning that uses features from the entire image maybe a better method to show the predictive benefit of medical images.

## Declaration of Competing Interest

The authors declare that they have no known competing financial interests or personal relationships that could have appeared to influence the work reported in this paper.
