# Supplementary material for: Radiomics biopsy signature for predicting survival in patients with spinal bone metastases (SBMs)
Source: Clin Transl Radiat Oncol. 2022 Jan 5;33:57–65. doi: 10.1016/j.ctro.2021.12.011 (PMC8777154; doi:10.1016/j.ctro.2021.12.011)
Supplement: Supplementary Data 1 [file mmc1.docx]

**Supplementary material**

***Figure 1.*** *PCA biplot showing individuals’ contributions to the first and second principal components by their survival status.*

*Patients 33 and 111 are somewhat different from the others and patient 11 is clearly different. A reexamination of the images of these patients reviled some artifacts for patient 11 and was excluded from further analysis.*


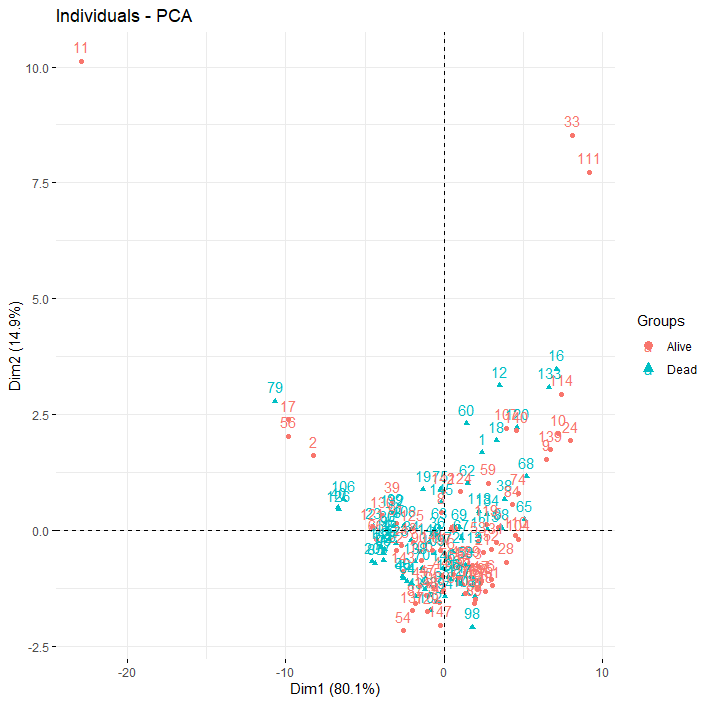


***Figure 2.*** *The* *scaled Schoenfeld residuals for each radiomics feature against the transformed time. The solid line is a smoothing spline fit to the plot, with the dashed lines representing a +/- 2-standard-error band around the fit.*


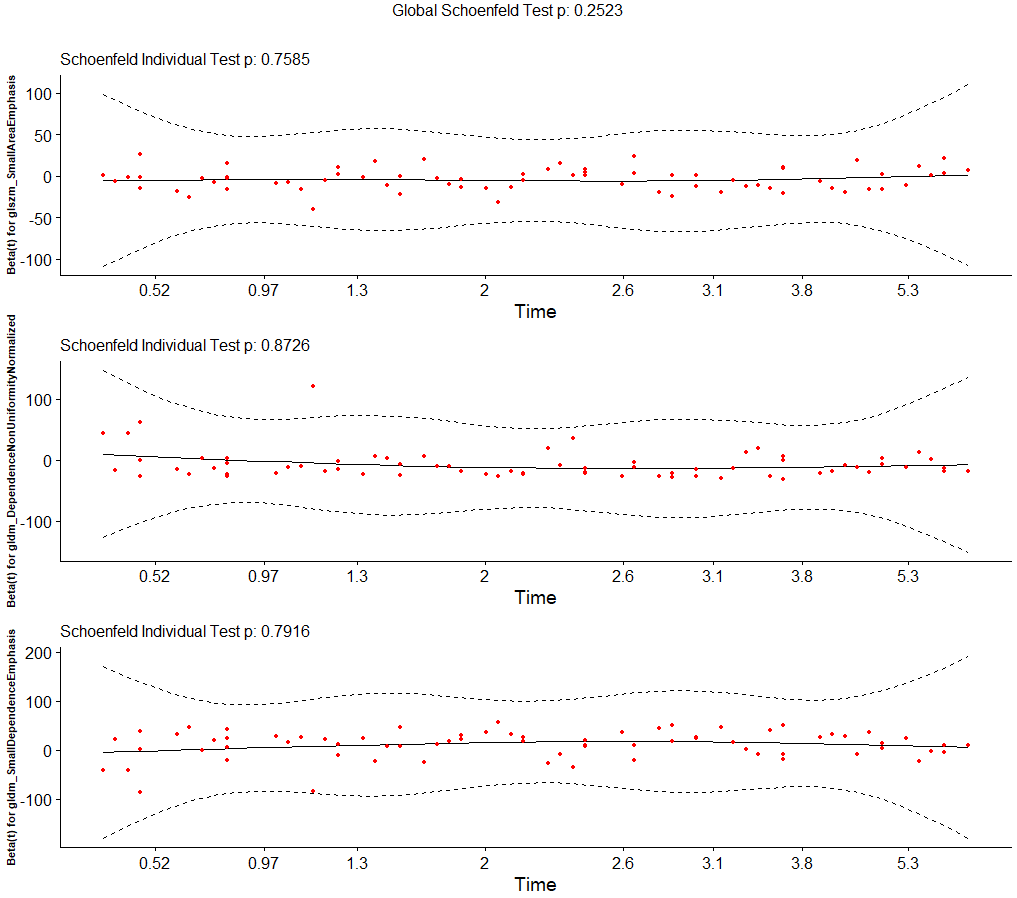


***Equation 1 and 2.***

| Prognostic Index (PI) Computation for the Rad and Clin Scores | |
| --- | --- |
| Clinscore = | +1.538 * Clinical profile (Moderate)  + 2.605 * Clinical profile (Unfavourable) **(1)** + 0.429 * WHO performance score (Self-Care)  + 0.875 * WHO performance score (Limited |
| Radscore = | - 0.415e01 * glszm Small Area Emphasis  + 0.108e02 * gldm Small Dependence Emphasis  **(2)**  - 0.716e01 * gldm Dependence Non-Uniformity Normalized |
